# Perceptions regarding the scope of practice of family doctors amongst patients in primary care settings in Nairobi

**DOI:** 10.4102/phcfm.v10i1.1818

**Published:** 2018-10-04

**Authors:** Gulnaz Mohamoud, Bob Mash, Mohamoud Merali, James Orwa, Megan Mahoney

**Affiliations:** 1 Department of Family Medicine, Aga Khan University Hospital, Kenya; 2 Division of Family Medicine and Primary Care, Stellenbosch University, South Africa; 3 Department of Counselling Psychology, Aga Khan University Hospital, Kenya; 4 Aga Khan University Hospital, Nairobi, Kenya; 5 Division of Primary Care and Population Health, Stanford University, United States of America

## Abstract

**Background:**

Primary care (PC) is the foundation of the Kenyan health care system, providing comprehensive care, health promotion and managing all illnesses across the lifecycle. In the private sector in Nairobi, PC is principally offered by the general practitioners, also known as family doctors (FDs). The majority have no postgraduate training. Little is known about how patients perceive their capability.

**Aim:**

To assess patients’ perceptions of the scope of practice of FDs working in private sector PC clinics in Nairobi and their awareness of the new category of family physicians (FPs) and the discipline of family medicine.

**Setting:**

Private sector PC clinics in Nairobi.

**Methods:**

A descriptive survey using a structured, self-administered questionnaire. Simple random sampling was used to recruit 162 patient participants.

**Results:**

Of the participants, 30% knew the difference between FPs and FDs. There was a high to moderate confidence that FDs could treat common illnesses; provide lifestyle advice; family planning (66%) and childhood immunisations (64%). In adolescents and adults, low confidence was expressed in their ability to manage tuberculosis (58%), human immunodeficiency virus (55%) and cancer (33%). In the elderly, there was low confidence in their ability to manage depression (55%), anxiety (57%), urinary incontinence (57%) and diabetes (59%). There was low confidence in their ability to provide antenatal care (55%) and Pap smears (42%).

**Conclusion:**

Patients did not perceive that FDs could offer fully comprehensive PC services. These perceptions may be addressed by defining the expected package of care, designing a system that encourages the utilisation of PC and employing FPs.

## Introduction

### Background

Forty years after the Alma-Ata Declaration that launched the primary health care (PHC) movement and which committed the World Health Organization (WHO) to tackle ‘politically, socially and economically unacceptable’ health inequities,^1^ major health inequities remain. According to a 2008 WHO report:

… it is necessary to invest in and renew the commitment to primary health care not only to promote health equity in developing countries, but also help to address the Millennium Development Goals, and now Sustainable Development Goals, at a population level.^2^

Primary health care has been defined as the sum of all elements of a health system meant to address basic health needs, including preventive care.^3^ The WHO further subdivides PHC into four main areas, that together, ensure a strong PHC system: universal health coverage, policy, leadership and governance, and primary care (PC).^3,4^ Health systems that effectively implement PHC achieve better results in comparison to those that focus more on a biomedical and hospital-based approach.^5^

Primary care is defined as the first contact with the health system and needs to be as accessible as possible. Primary care is characterised as being long-term and patient-centred; that is, both comprehensive and coordinated with other levels of care in the context of both family and community.^6^

In European countries, most reforms aim to create health care systems with efficiently organised PC services as their foundation. In the Netherlands and the United Kingdom, general practitioners (GPs) function as the PC providers and gatekeepers to the health system.^7^

Primary care delivery in sub-Saharan Africa (SSA) has delivered selected programmes for diseases such as human immunodeficiency virus (HIV), tuberculosis (TB) and malaria, but has been criticised for not being comprehensive and being poorly resourced in terms of equipment, medications and delivered by low-level and poorly trained health care workers.^2,8,9^ Sub-Saharan Africa countries face many challenges with regards to the accessibility and affordability of high-quality PC. Whilst the WHO encourages the promotion of comprehensive PC, it is still an emerging concept in SSA.^10^

A sustained focus on and attention to PC in the public sector in Kenya has resulted in a doubling of utilisation over a 10-year period.^11^ This was achieved through increased staffing, provision of essential commodities, elimination of cash payments and introduction of free maternal services.^11^ Two-thirds of all consultations in PC now occur in the public health care sector and health equity has improved.^11^ Clinical officers (mid-level clinicians) and GPs are the main PC providers in both the private and public sector. Doctors are essential contributors to strengthening the PHC system in both the public and private sectors.^12^

Little is known in Kenya about the performance of PC, particularly in terms of access, comprehensiveness, continuity, coordination, people-centeredness and quality of care. Health information systems struggle to collect, analyse, interpret and use data for improvement.^5^ Most studies have focused on hospital-based care in the public sector.^13^

Nairobi, the capital city of Kenya, is home to approximately 3 million people and is the largest city in Kenya.^14^ Primary care in the private sector is provided mainly by GPs, hereafter referred to as family doctors (FDs), most of whom have not received postgraduate training in family medicine (FM).

Family medicine trains doctors to work in PC and the broader district health services, but in Kenya is still in its nascent stage with no more than 100 registered family physicians (FPs). In contrast, the number of GPs total around 3400. As FPs, with four years of postgraduate training, enter the health system, there may be a mismatch between their more comprehensive competencies and public expectations of the scope of PC practice based on untrained FDs. Little is known about how the patients view the competence of the existing FDs in private practice, the range of services that they offer and whether they are aware of the new discipline of FM and how this could further benefit services.

A pilot study carried out in Nairobi demonstrated a higher recognition of the term ‘family doctor’, amongst patients attending a PC clinic, which led to the use of this term in this study. However, there was less understanding of what FDs do, what conditions they could treat or procedures they could perform. There is very little research on the services offered by the FDs in the private sector in PC in Kenya and even less from the patient’s perspective. Understanding the current perceptions of patients regarding the scope of services offered by the FDs in Kenya, is a crucial step in promoting, marketing and planning the delivery of PC services.

This study aimed to assess patient’s perceptions of the scope of practice of FDs working in private sector PC clinics in Nairobi and their awareness of the new category of FPs and the discipline of FM.

## Methods

### Study design

This was a cross-sectional descriptive survey using a structured self-administered questionnaire.

### Setting

The Kenyan health system consists of three main categories of service providers: public, private non-profit and private for-profit organisations. The government operates 41% of health facilities in the public sector, whilst the private for-profit sector operates 43% of health facilities and is becoming more prominent. Private clinics of varying complexity exist in most major urban centres.^15^

There were 13 PC clinics associated with the tertiary care private hospital within the city of Nairobi at the time of the study. These clinics were run by FDs and offered health promotion, preventive and curative services to all age groups. The staff in each clinic included a FD, registered nurse, laboratory technician and pharmacist. On an average, 35 patients were seen at these clinics per day. Almost all of them were covered by private medical insurance.

### Study population and sampling strategy

A non-stratified Fisher’s sample size calculation was based on: an expected proportion of 50%, as there was no previous data to estimate the expected proportions across multiple variables, a margin of error of 5% and a study population of 280 which was the sum of the daily average number of patients seen in the eight selected facilities. This resulted in a minimum sample size of 162 participants.

The study included eight clinics with the highest workload, of which three clinics were located in the centre of Nairobi and five in the broader metropolitan area.

The proportion of the sample selected from each clinic was based on the proportion of the total workload (average daily headcount) seen in each clinic in the study. In each clinic, simple random sampling was used to recruit the participants. The study population consisted of adult patients (18 years and above) attending the clinics. Those who were too sick or those who could not provide their own consent were excluded from the study.

### Data collection

Data were collected using a structured self-administered questionnaire. The questionnaire was developed by the researcher, based on the common conditions seen in the Nairobi context and services usually provided by PC. As there was no defined package of care for the PC services offered in the private sector, items were generated by G.M. (an experienced FP) and M.M. (head of the FM department) and the content and construct of the questionnaire was further validated by a FD and a medical psychologist, all of whom had research expertise and work experience in PC. The questionnaire was piloted in a PC clinic also associated with the same tertiary care hospital to assess its acceptability, feasibility and ease of understanding in the study population. This clinic did not form part of the main study. The feedback from the pilot was used to adjust the final questionnaire.

All the patients attending the clinic spoke English and consultations and treatment at all the chosen clinics were conducted in English, hence the choice of language. Literacy level in Nairobi is measured at 85%.^14^

The questionnaire captured information on demographics and patients’ perceptions of whether FDs could offer clinical services for different age groups, acute and chronic conditions, disease prevention and health promotion.

As a secondary objective, patients were also asked about their awareness of FM and the new cadre of FPs. Patient perception was defined as patient’s awareness of the conditions, preventive health care services and life style advice that the FD could treat or carry out.

### Data analysis

Data were captured in MS Excel, checked for errors or omissions and then analysed in Strata version 12. All variables were categorical and were analysed descriptively using frequencies and percentages. The patients’ perception as to whether the FD could offer a service was categorised into high (≥ 80%), moderate (60% – 79%) and low (< 60%).

## Ethical consideration

The study was approved by the tertiary care hospital’s Research and Ethics Committee (research protocol no. 2014/REC-50(v3)) and by the National Commission for Science, Technology and Innovation (permit no. NACOSTI/P/15/1304/5166).

## Results

### Demographics

The socio-demographic characteristics of the 162 participants are presented in Table 1. Overall, there were 75 (46%) men and 87 (54%) women. The majority were married, had children, were of working age, employed, with tertiary education (83%) and lived in Nairobi.

**TABLE 1:** Socio-demographic characteristics of participants.

Characteristics	Male *N* = 75		Female *N* = 87		Total *N* = 162	
	*n*	%	*n*	%	*n*	%
**Clinic**						
Buruburu	12	16.0	8	9.2	20	12.3
Doonholm	8	10.7	7	8.0	15	9.3
Kikuyu	15	20.0	15	17.2	30	18.5
Ongata Rongai	5	6.7	10	11.5	15	9.3
Ridgeways	7	9.3	6	6.9	13	8.0
Ruaka	7	9.3	14	16.1	21	13.0
Syokimau	18	24.0	23	26.4	41	25.3
T-mall	3	4.0	4	4.6	7	4.3
**Age (years)**						
18–30	28	37.3	44	50.6	72	44.4
31–45	35	46.7	37	42.5	72	44.4
46–60	11	14.7	5	5.7	16	9.9
> 60	1	1.3	1	1.1	2	1.2
**Occupation**						
Self-employed	10	13.3	23	26.4	33	20.4
Employed	51	68.0	47	54.0	98	60.5
Student	10	13.3	13	14.9	23	14.2
Retired	4	5.3	4	4.6	8	4.9
Education						
O-level	7	9.3	6	6.9	13	8.0
A-level	4	5.3	10	11.5	14	8.6
University and/or college	64	85.3	71	81.6	135	83.3
**Marital status**						
Single	21	28.0	29	33.3	50	30.9
Married	50	66.7	57	65.5	107	66.0
Other	4	5.3	1	1.1	5	3.1
**Residential area**						
Nairobi	52	69.3	55	63.2	107	66.0
Nairobi metropolitan	23	30.7	32	36.8	55	34.0

T-Mall, tuskys mall; O-level, ordinary level; A-level, advanced level.

### Difference in perception between family doctor and family physician

**FIGURE 1:**
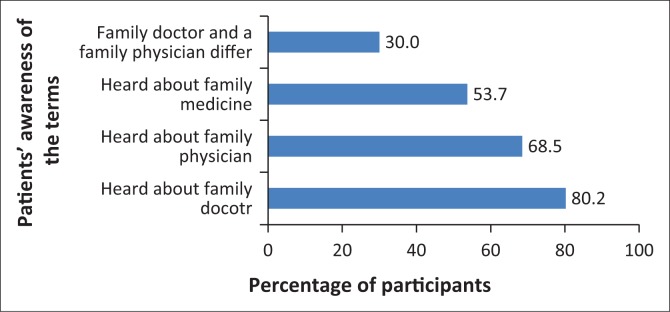
Patients’ awareness of family physicians, family doctors and family medicine.

**FIGURE 2:**
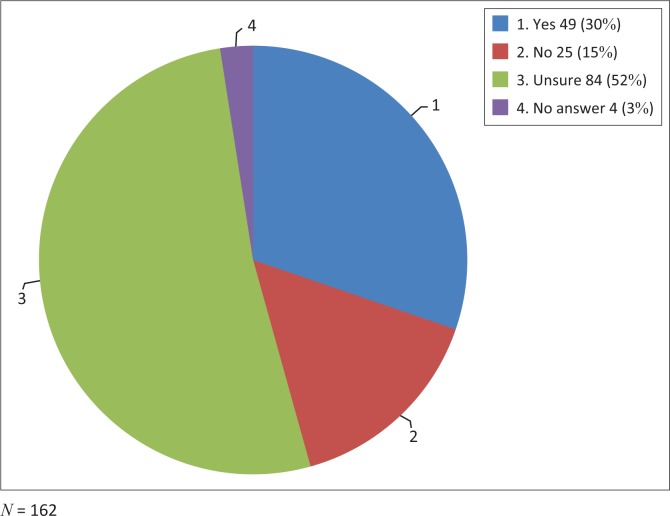
Patients understanding that a family doctor and a family physician differ.

Most respondents had heard of the terms FP, FD and FM (Figure 1). In all, 49 (30%) stated that there was a difference between a FD and a FP (Figure 2).

### Perceptions regarding the scope of practice of a family doctor

Most respondents believed that FDs were able to treat people across the lifecycle, although the perception was slightly less for small babies and the elderly (Table 2). Table 2 also presents the patients’ perceptions regarding the different diseases a FD could treat across different age groups.

**TABLE 2:** Perception of the diseases a family doctor can treat across different age groups.

Type of disease	Male		Female		Total	
	*n*	(%)	*n*	(%)	*n*	(%)
**Babies and children ≤ 12 years**							
Diarrhoea	61	81.3	72	82.8	133	82.1
Fever	68	90.7	76	87.4	144	88.9
Cough	67	89.3	76	87.4	143	88.3
Flu	66	88.0	74	85.1	140	86.4
Vomiting	66	88.0	74	85.1	140	86.4
Sore throat	59	78.7	71	81.6	130	80.2
Pneumonia	55	74.3	61	70.1	116	72.0
Ear infections	50	66.7	60	69.0	110	67.9
Eye infections	45	60.0	58	67.4	103	64.0
Asthma	48	64.0	57	65.5	105	68.0
Skin diseases	43	61.4	48	57.8	91	59.5
**Adolescents 13 years–17 years**							
Cough	70	93.3	79	91.9	149	92.5
Malaria	67	89.3	78	90.7	145	90.1
Diarrhoea	68	90.7	79	91.9	147	91.3
Headache	70	93.3	80	93.0	150	93.2
Flu and cold	68	90.7	78	90.7	146	90.7
Skin diseases	48	64.0	61	70.9	109	67.7
Diabetes	44	58.7	52	61.9	96	60.4
HIV infection	41	54.7	45	52.9	86	53.8
TB infection	44	58.7	47	54.7	91	56.5
STI	50	66.7	56	65.1	106	65.8
Asthma	49	65.3	59	69.4	108	67.5
Injuries	63	84.0	70	82.4	133	83.1
Cancer	24	32.0	27	31.4	51	31.7
**Adults 18–60 years**							
Cough	70	93.3	81	93.1	151	93.2
Malaria	67	89.3	80	93.0	147	91.3
Diarrhoea	68	90.7	80	92.0	148	91.4
Headache	70	93.3	81	93.1	151	93.2
Flu and cold	69	92.0	82	94.3	151	93.2
Diabetes	53	70.7	58	68.2	111	69.4
High blood pressure	53	70.7	64	4.4	117	72.7
HIV infection	40	53.3	50	58.8	90	56.3
TB infection	43	57.3	52	60.5	95	59.0
STI	54	54.0	63	73.3	117	72.7
Asthma	50	67.6	63	73.3	113	70.6
Injuries	64	85.3	67	77.9	131	81.4
Chest pain	37	49.3	46	54.1	83	51.9
Cancer	24	32.0	32	37.6	56	35.0
Thyroid problems	36	48.0	43	50.0	79	49.1
**Elderly ≥ 61 years**							
Arthritis	43	59.7	52	59.8	95	59.7
Joint pains and/or stiffness	47	66.2	61	70.1	108	68.4
Injuries from falling	52	73.2	62	71.3	114	72.2
Depression	41	56.9	47	54.0	88	55.3
Anxiety	41	56.9	49	56.3	90	56.6
Urinary incontinence	36	50.0	54	62.1	90	56.6
Diabetes	41	56.9	52	60.5	93	58.9
High blood pressure	48	66.7	60	69.8	108	68.4
Sleep problems	43	59.7	54	62.1	97	61.0
Asthma	43	59.7	58	67.4	101	63.9
Skin diseases	46	63.9	59	67.8	105	66.0
Thyroid problems	35	48.6	43	49.4	78	49.1

HIV, human immunodeficiency virus; STI, sexually transmitted infections; TB, tuberculosis.

In children below 12 years, patients expressed high confidence in the FDs’ ability to treat diarrhoea and upper respiratory tract infection; moderate confidence in their ability to manage lower respiratory tract infections, asthma, ear and eye infections, and low confidence to treat dermatological problems.

In adolescents, there was a high level of confidence that FDs were able to treat cough, malaria, diarrhoea, headache, upper respiratory tract infections and injuries. There was moderate confidence in the treatment of skin diseases, diabetes, sexually transmitted infections (STIs) and asthma. There was a low level of confidence for the treatment of HIV, TB and cancer.

In adults, there was a high level of confidence that a FD could treat common conditions such as cough, malaria, diarrhoea, headache, upper respiratory tract infections and injuries; a moderate level of confidence was expressed regarding the treatment of chronic conditions such as diabetes, high blood pressure, asthma as well as STIs. There was low confidence expressed in their ability to treat HIV, TB, chest pain, cancer and thyroid disease.

Regarding the elderly, patients had moderate level of confidence that FDs could treat joint pains and/or stiffness, injuries from falls, high blood pressure, sleep problems, asthma and skin diseases, but low level of confidence in treating depression, anxiety, urinary incontinence, diabetes and thyroid problems.

The patients had moderate confidence that the FD could offer services like childhood immunisation, adult vaccination and family planning and low levels of confidence in antenatal care, cervical cancer screening and circumcision (Table 3).

**TABLE 3:** Perception of family doctors offering preventative health care services (*N* = 162).

Variables	Total	
Type of specialised services	*n*	(%)
Antenatal care	88	55.0
Carry out Pap smear tests	67	41.9
Family planning services	105	65.6
Childhood immunisation	103	64.0
Adult vaccinations	108	67.0
Circumcision	92	57.1

Regarding lifestyle issues, patient’s had moderate confidence that a FD could advise on obesity and weight loss, on nutrition in general, behaviour change counselling for tobacco smoking and alcohol and low level of confidence was expressed regarding end-of-life issues (Table 4).

**TABLE 4:** Perception of family doctors offering lifestyle advice and counselling (*N* = 162).

Variables	Total	
Type of lifestyle	*n*	%
Healthy diet for chronic diseases	128	80.0
Childhood nutrition	125	78.1
Obesity and weight loss	116	72.5
Psychosocial counselling	121	76.1
End-of-life issues	89	56.3
Alcohol and smoking	103	64.4

## Discussion

This study showed that patients had limited expectations of the services offered by FDs in the private sector PC in Nairobi. They had confidence in the FDs’ ability to treat common minor illnesses and non-communicable diseases, as well as underlying risky behaviours such as tobacco smoking and harmful alcohol use. They were not fully convinced by the FDs’ ability to treat communicable diseases, emergencies, pregnant women, skin conditions, cancer or to provide preventive care such as cervical cancer screening and immunisations. They also lacked confidence in the FDs’ ability to treat small babies and the elderly.

Whilst our study showed positive confidence in FDs’ abilities to treat common illnesses, there were gaps that indicated the need for more awareness on the role of FDs, as well as empowerment of the FDs to reduce them. Studies elsewhere concur that the ability of FDs to treat common conditions and to counsel patients on lifestyle issues are important in saving patients’ time, cost and in preventing disease. However, their results also showed gaps in the patient’s perception of what FDs could deliver in this context compared to what would be expected elsewhere.^16^

The patients’ perceptions of the scope of practice may have been influenced, not only by the capability of the FDs, but also by the private medical system itself, which does not automatically offer all the PC services. Medical insurance may enable direct access to general specialists such as paediatricians and obstetricians for common illnesses or routine antenatal care that could be competently provided by FDs in PC. Patients may also prefer care offered by a specialist with regards to non-communicable diseases and specialists may resist their business being taken over by PC. This emphasises the need for the FDs and the specialists to work together not only to maximise efficiency of care but also for the benefit of the patient.^17^

There may not be an incentive for PC to act as a gatekeeper to the health system in the private sector, whilst in the public sector; gatekeeping is needed to make the system more efficient and equitable. Health systems that allow patients to directly access specialist care may be less efficient in the use of resources, cause more harm and patients can be less satisfied.^18,19^ A study by Starfield et al. showed that ‘the supply of PC physicians was significantly associated with lower all-cause mortality, whereas a greater supply of speciality physicians was associated with higher mortality’.^6^ However, in a system with untrained FDs, a lack of trust in their capabilities might drive patients to consult hospital specialists.^17,19^

The lack of confidence in FDs’ ability to manage children could be explained by the fact that specialist paediatric care is initiated in all private hospitals immediately from the time of birth. The general tendency is for the parents to continue with this care from a paediatrician along with childhood immunisation. A study also confirmed the tendency by FDs in private practice to refer patients to the public sector for immunisation because of the high costs involved, thereby diminishing their role in this important activity.^20^ In the public sector in Kenya, antenatal services, delivery, postnatal care and childhood immunisations are all provided free of cost. In addition, private insurance does not usually fully cover costs related to these areas of health need. Hence, there is fairly high usage of these services from the public health facilities which may explain our findings whereby patient’s expressed low confidence in FDs’ ability to manage women’s health.

A similar situation exists with regard to care for HIV, TB and STIs. Care is provided free for these conditions in the public sector; hence, most patients are managed in this setting. Family doctors, therefore, may become deskilled in managing these conditions. In addition, patients can also access care from infectious disease specialists in the private sector and bypass PC.

Emergency care is usually provided at emergency centres within the hospital environment. This could have contributed to the low perception about FDs treating emergencies such as chest pain.

Cancer treatment is typically within the domain of the oncologist, although shared care with a FD is recommended to bring a more holistic and family-orientated approach.^21,22^ Not surprisingly, therefore, the perception in this study is similar to others that FDs were not very capable at end-of-life counselling and cancer care.^23,24^ FM and palliative care have been intimately linked in many health systems.

The perception that FDs were less capable at managing the elderly could be because of the complexity of multi-morbidity in the elderly, and the challenges of multiple cognitive, medical and social issues. Also, reliance on specialist care, along with a gap in the training of the FDs in elderly care could have contributed to this perception. In addition, the number of elderly patients seen in PC in Nairobi are few, as most of them reside in their rural homes. In this study, only two of the respondents were above the age of 60 years. Although caring for the elderly in PC can be fulfilling and rewarding, it is also complex, difficult, heart breaking and time-consuming, more so with the addition of geriatric mental disorders.^25,26^

Although the scope of practice of FDs in the private sector may be shaped by the private health system itself, there may also be issues with the capability of FDs. Doctors with a basic medical degree often have gaps in their undergraduate training that reflects the tertiary hospital teaching setting. Care of common conditions seen in general practice may be omitted, particularly skin, eye, ear and nose problems, which are sub-specialities in the tertiary hospital environment.

Tertiary hospital training may also reduce exposure to undifferentiated problems, psychosocial issues and the health needs of the community. Hence, graduates may not be well prepared for PC.

Postgraduate training in FM is a recent initiative in Kenya, and there are now five training programmes (four in the public sector and one in the private sector). Postgraduate training in FM prepares doctors to become FPs with a more comprehensive set of competencies for PC and district hospital settings.^10^ Family physicians are also trained to support the development of comprehensive PC that is equitable, accessible, continuous, patient-centred and holistic.^10^ The number of FPs in the health system of Kenya is still very low, therefore, it was not surprising to find that patients were not fully aware of this new cadre of specialists.

Patients’ perceptions on the scope of practice may be shaped, not only by their experience, but also by shared beliefs in their families or communities on the services offered by the PC clinics. This may be based on past or other experiences of the health system, media stories or other factors. This may merit further studies.

### Strengths and limitations of the study

Because of the absence of a defined package of care, some diseases or services may not have been fully captured in the questionnaire. It is likely that the patients attending the eight clinics shared similar socio-economic and demographic characteristics to patients seen in the other PC clinics of the same hospital. Hence, the findings could be generalised to include patients attending all the PC clinics associated with this particular private tertiary care hospital, but cannot be generalised to other private and public health facilities.

### Implications or recommendations

The FDs need to upgrade their skills for the management of different conditions, which will help them to improve the care provision. This will positively affect patient’s perceptions regarding their capabilities in handling various conditions and performing certain procedures.

With the development of FM in Kenya, the FDs could be promoted to FPs after 4 years of postgraduate training. The FPs would bring a more consistent and comprehensive set of competencies to the practice. Patients, however, are not yet aware of the new speciality and might not change their perceptions or behaviour easily in a system that incentivises access to specialists. If patients have low expectations of PC, then the tendency to bypass clinics for anything but minor illness will be high.

The beliefs of patient’s in this study are consistent with this type of health-seeking behaviour, which may change over time as the patients become more aware of the scope and services provided by the FPs.

The health system provision would need to be changed and reorganised if highly trained and competent FPs are to be placed in the PC clinics in order for the patients to fully benefit.

Here the managed care organisations might encourage patients to access specialist care through FPs because it is more cost-effective, accessible, comprehensive and patient-centred.

Patients’ lack of confidence in a comprehensive PC service could also be addressed by clearly defining the expected package of care and ensuring that patients’ expectations and health-seeking behaviour are modified through effective communication strategies. The findings of the study may have been different if it had been carried out in the rural setting where access to specialist care is more difficult. Patients’ ideas could also be further explored in qualitative studies.

## Conclusion

Patients did not perceive that FDs could offer a fully comprehensive PC service and were not very clear about the difference between a FD and a FP.

They believed that FDs could handle common illnesses, common chronic conditions and counsel on lifestyle change. They were less convinced that FDs could offer care for communicable diseases, such as HIV and TB, to pregnant women, small babies, the elderly, manage skin problems and offer some forms of prevention such as immunisations and cervical cancer screening. Perceptions may be addressed by defining the expected package of care, designing a system that encourages utilisation of PC and ensuring that care is offered by competent generalists. One way of ensuring this is to employ FPs in the PC clinics.
